# A novel lactate metabolism-related signature predicts prognosis and tumor immune microenvironment of breast cancer

**DOI:** 10.3389/fgene.2022.934830

**Published:** 2022-09-07

**Authors:** Zhihao Zhang, Tian Fang, Yonggang Lv

**Affiliations:** ^1^ Department of Thyroid Breast Surgery, Xi’an NO. 3 Hospital, The Affiliated Hospital of Northwest University, Xi’an, China; ^2^ Department of Medical Oncology, Cancer Center, West China Hospital of Sichuan University, Chengdu, China

**Keywords:** lactate metabolism, breast cancer, immune infiltration, prognosis, gene

## Abstract

**Background:** Lactate, an intermediate product of glycolysis, has become an essential regulator of tumor maintenance, development, and metastasis. Lactate can drive tumors by changing the microenvironment of tumor cells. Because of lactate’s important role in cancer, we aim to find a novel prognostic signature based on lactate metabolism-related genes (LMRGs) of breast cancer (BC).

**Methods**: RNA-sequencing data and clinical information of BC were enrolled from The Cancer Genome Atlas (TCGA) and Gene Expression Omnibus (GEO) database. We obtained LMRGs from the Molecular Signature Database v7.4 and articles, and then we compared candidate genes with TCGA data to get differential genes. Univariate analysis and most minor absolute shrinkage and selector operator (LASSO) Cox regression were employed to filter prognostic genes. A novel lactate metabolism-related risk signature was constructed using a multivariate Cox regression analysis. The signature was validated by time-dependent ROC curve analyses and Kaplan–Meier analyses in TCGA and GEO cohorts. Then, we further investigated in depth the function of the model’s immune microenvironment.

**Results:** We constructed a 3-LMRG-based risk signature. Kaplan–Meier curves confirmed that high-risk score subgroups had a worse prognosis in TCGA and GEO cohorts. Then a nomogram to predict the probability of survival for BC was constructed. We also performed Gene Ontology (GO) enrichment analysis and Kyoto Encyclopedia of Genes and Genomes (KEGG) pathway function analysis. The function analysis showed that the lactate metabolism-related signature was significantly related to immune response. A significant correlation was observed between prognostic LMRGs and tumor mutation burden, checkpoints, and immune cell infiltration. An mRNA–miRNA network was built to identify an miR-203a-3p/LDHD/LYRM7 regulatory axis in BC.

**Conclusion:** In conclusion, we constructed a novel 3-LMRG signature and nomogram that can be used to predict the prognosis of BC patients. In addition, the signature is closely related to the immune microenvironment, which may provide new insight into future anticancer therapies.

## Introduction

Breast cancer (BC) is the most common cancer among women, which seriously threatens women’s health. The current risk stratification of BC is mainly based on clinicopathological factors, including tumor stage, tumor size, lymph node status, and molecular subtypes (Ding et al., 2021). However, the high heterogeneity of BC causes the current stratification to fail to provide proper treatment and prognostic information, leading to inadequate or excessive treatment (Wang et al., 2020). Therefore, it is necessary to develop novel and credible prognostic markers to provide more accurate stratification for BC, which can guide clinicians to make better individualized treatment decisions.

In recent years, tumor metabolism has received increasing attention. In particular, there has been increased interest in the “Warburg effect” (Goodwin et al., 2019). Despite normoxic conditions, tumor cells will still use glycolysis, which leads tumor tissues to have more lactate accumulation than normal tissues. Lactate promotes tumor cell migration, aggregation, and immune escape by regulating the microenvironment. In addition, a high concentration of lactate can directly kill immune cells (Warburg, 1956). Lactate can regulate the function of immune cells in tumors, thereby establishing an immunosuppressive microenvironment to promote tumor progression (Feichtinger and Lang, 2019). Therefore, lactate plays an essential role in tumorigenesis and proliferation in tumor cells. But there is a paucity of lactate metabolism-related signatures of BC. Accordingly, we aimed to establish a lactate metabolism-related signature for BC.

In our study, we analyzed RNA-seq and clinical information in The Cancer Genome Atlas (TCGA) dataset to filter differentially expressed LMRGs between breast cancer and normal tissues, and further extracted prognostic genes to construct a lactate metabolism-related prognostic signature. The function enrichment analysis indicated that the module was significantly related to immune response. Therefore, we further analyzed the relationship between lactate-related prognostic genes and the immune microenvironment. The results show that this model can not only predict survival but also stratify patients and low-risk BC patients who are often more sensitive to immunotherapy.

## Materials and methods

### Datasets and preprocessing

In our study, the transcriptome RNA-sequencing data and clinical information of BC were enrolled from The Cancer Genome Atlas (TCGA) (https://portal.gdc.cancer.gov/)*,* including 104 normal tissues and 1,078 tumor tissues. The gene expression matrix and clinical data for the validation cohort were obtained from the Gene Expression Omnibus database (GEO, https://www.ncbi.nlm.nih.gov/geo/). GSE53031 includes 197 tumor samples with breast cancer. Data analysis was performed with the R (version 4.1.0) and R Bioconductor packages.

### Preprocessing and analysis of lactate metabolism-related gene expression data

A total of two gene sets related to lactate metabolism (lactate metabolic process and lactate transmembrane transport) were acquired from the Molecular Signature Database v7.4 (MSigDB; https://www.gsea-msigdb.org/gsea/msigdb) (Liberzon et al., 2011). Moreover, we extracted the other three genes related to lactate metabolism by browsing the articles. We found differentially expressed genes (DEGs) in BC and normal tissues through the “limma” R package with the specific criteria of FDR <0.05 and | log2FC |≥1 in TCGA cohort. The differentially expressed LMRGs were exhibited by the heatmap and gene ranking dot plot.

### Establishment of a lactate metabolism-related gene prognostic signature

We obtained lactate metabolism genes significantly associated with prognosis by generating a univariate analysis. Subsequently, the least absolute shrinkage and selector operator (LASSO) Cox regression (R package “glmnet”) was performed to prevent the overfitting problem (Goeman, 2010). Based on these prognostic LMRGs, we constructed a prognostic model by developing multivariate Cox regression. The risk score was based on the specific formulate “Riskscore = gen1 * coef1 + gen2 * gen2 + … + genx * coefx.” Then the samples were divided into high and low groups according to the median score. The overall survival between the two risk subgroups was compared by Kaplan–Meier analysis with the log-rank test. The “survivalROC” R package generated the time-dependent curve analysis to assess the predictive accuracy of the LMRG prognostic signature.

### Independent prognostic analysis of the signature

We accessed the BC patients’ clinical information (age, stage, pT, pN, and pM). These variables were analyzed in combination with the risk score by conducting univariate and multivariable Cox regression. We also compared the clinical information with prognostic LMRGs. Subsequently, we developed a nomogram to predict the probability of survival.

### Functional enrichment and immune analysis between the two risk groups

We divided BC patients into high- and low-risk subgroups based on the median scores. We compared the two risk groups to extracted DEGs with specific criteria (|log2FC|≥1 and FDR <0.05). Based on these candidate DEGs, Gene Ontology (GO) enrichment analysis and Kyoto Encyclopedia of Genes and Genomes (KEGG) pathway analyses were generated by applying the “clusterProfiler” package. Then the “gsva” R package was developed to conduct the ssGSEA to compute the scores of infiltrating immune cells and immune-related pathways.

### Immune infiltration, checkpoint, and tumor mutation burden analysis

We used the Tumor Immune Estimation Resource (TIMER; https://cistrome.shinyapps.io/timer/) to analyze the relationship between prognostic genes and immune cell infiltration. The “SCNA” function of the TIMER database was used to explore the somatic copy number alterations (SCNA) of prognostic genes and their effect on the infiltration levels of immune cells. Moreover, immune checkpoints (PD-L1 and CTLA-4) and tumor mutation burden (TMB) were further explored in the risk models.

### MRNA–miRNA network construction

We developed an mRNA–miRNA network to further explore the potential function of LMRGs in BC. TargetScan (http://www.targetscan.org/) was used to predict the miRNA targets binding to the LMRGs. We examined the expression and features of this miRNA target using TCGA BC datasets.

### Statistical analysis

Univariate and multivariate Cox regression analyses were conducted to identify independent prognostic survival factors. The model’s predictive value for OS was assessed by performing Kaplan–Meier analysis. The time-dependent ROC curves evaluated the predictive accuracy of this model. We conducted all data analysis with the R (version 4.1.0) and R Bioconductor packages. The *p*-value < 0.05 was regarded statistically significant.

## Result

### Identification of lactate metabolism-related differentially expressed genes in The Cancer Genome Atlas

**FIGURE 1:**
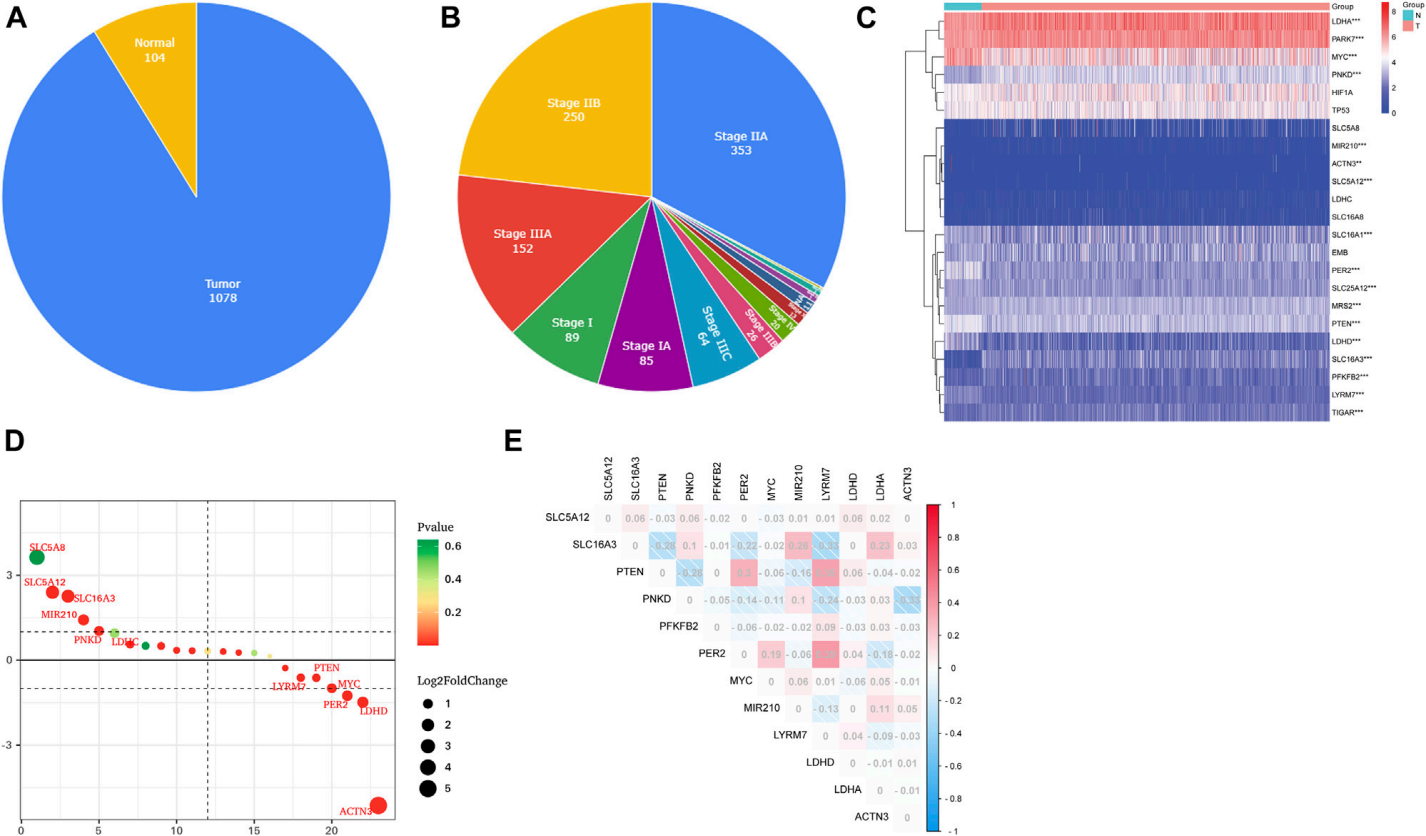
Characteristics of TCGA BC patients and expressions of the LMRGs. **(A,B)** Clinicopathological of all patients included in this study. **(C)** Heatmap (blue: low expression level; red: high expression level) of the LMRGs between the normal (N, brilliant blue) and tumor tissues (T, red). *p*-values were shown as *p < 0.05; **p < 0.01; and ***p < 0.001. **(D)** Gene ranking dot plot (The redder the color, the closer the *p*-value is to zero; the bigger the circle, the taller the | log2FC |). **(E)** Interaction analysis among the 12 LMRGs.

The detailed flowchart is shown in Supplementary Figure S1. We obtained 1,182 breast RNA-sequencing and clinical data from TCGA database. Those samples’ characteristics are shown in Figures 1A,B. To identify genes related to lactate metabolism, we selected two pathways related to lactate metabolism through the MSigDB database and extracted 22 related genes. We found three additional genes (*LYRM7, MYC,* and *PTEN*) by reading articles related to lactate metabolism (Le et al., 2010; Invernizzi et al., 2013; van der Mijn et al., 2017). DEGs were defined with the specific criteria of FDR <0.05 and | log2FC |≥1. Finally, 12 LMRGs were identified (Figures 1C,D), among which *LDHA, MIR210, PFKFB2, PNKD, SLC16A3,* and *SLC5A12* were upregulated, and *ACTN3, LDHD, LYRM7, MYC, PER2,* and *PTEN* were downregulated; the characteristics are listed in Supplementary Table S1. In addition, we further studied the interrelationships between these 12 LMRGs (Figure 1E).

### Construction and assessment of a prognostic model based on selected lactate metabolism-related genes

**FIGURE 2:**
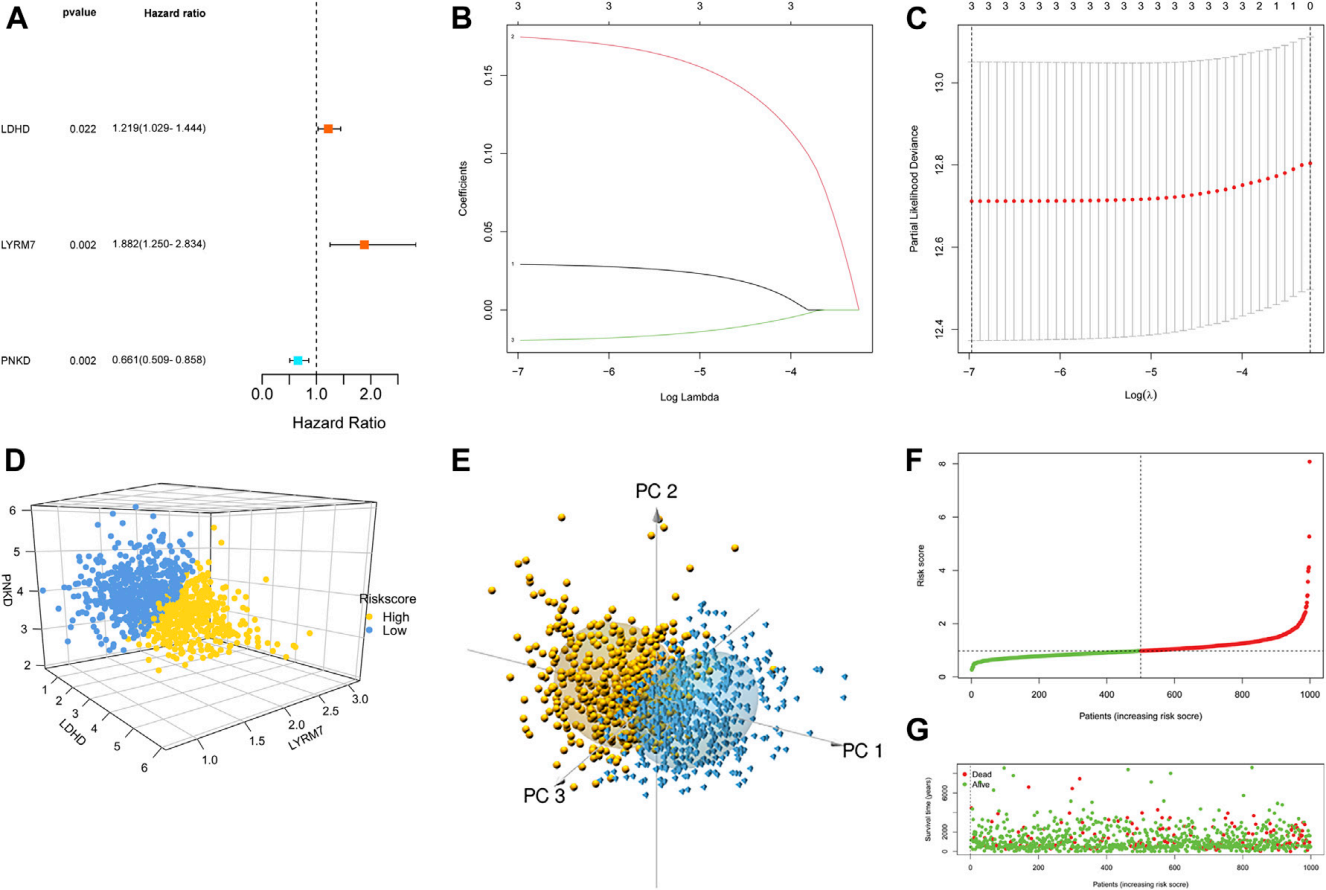
Construction of the LMRG prognostic signature. **(A)** Univariate Cox regression analysis of 12 LMRGs, and three genes with *p* < 0.05. **(B,C)** LASSO regression analysis. **(D)** Two risk score groups were separated based on the expression of three genes. **(E)** PCA plot for OCs based on the risk score. **(F,G)** Risk score distribution and survival status of BCs based on the risk score.

**FIGURE 3:**
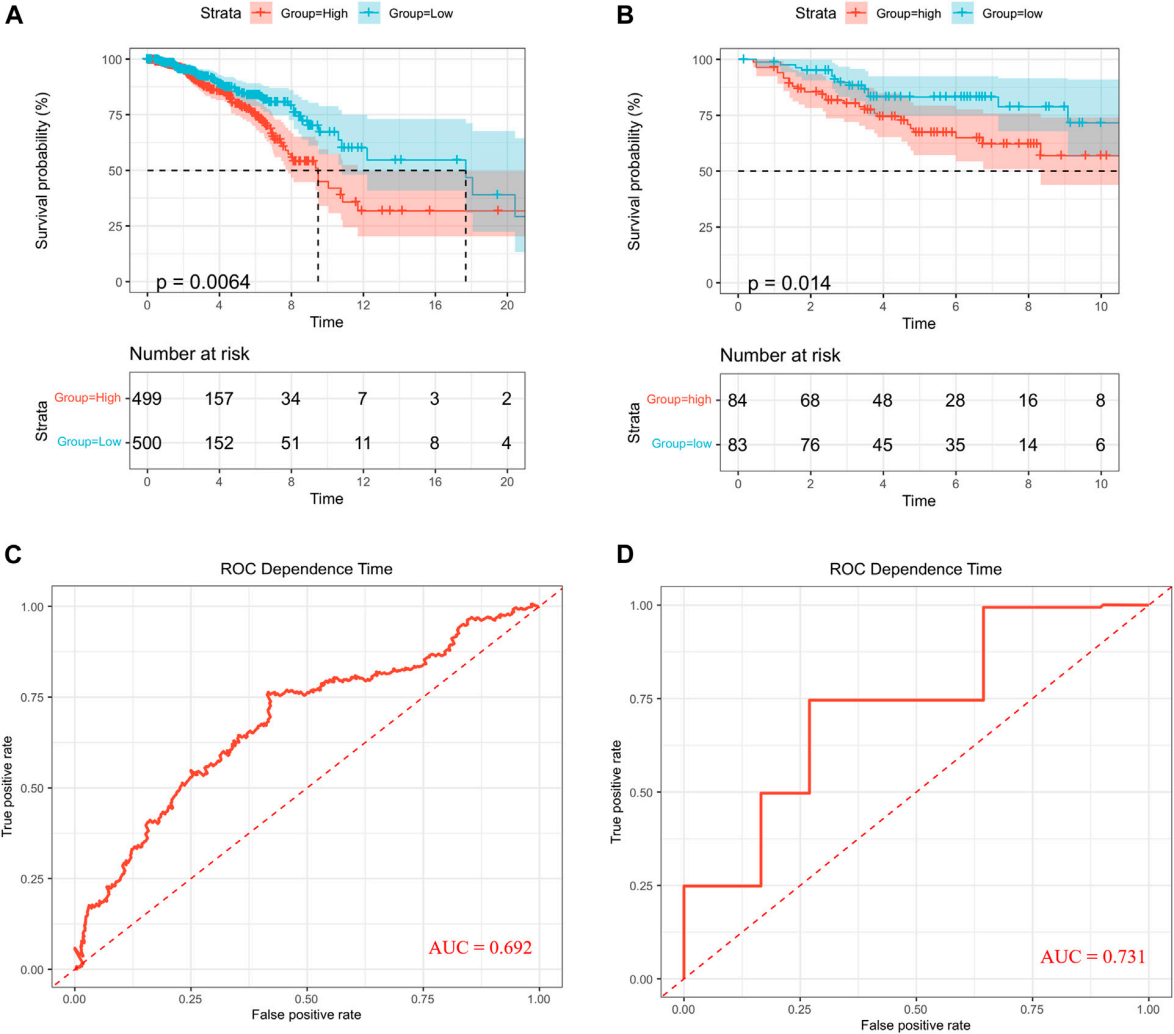
Survival analysis. Kaplan–Meier curves in TCGA **(A)** and GEO **(B)**. ROC curves in TCGA **(C)** and GEO **(D)**.

First, 12 LMRGs correlated with OS were identified by performing a univariate Cox regression; three genes that meet the criteria of *p* < 0.05 have significant prognostic value, one gene (PNKD) performed a protective effect with HRs<1, and two genes (LDHD and LYRM7) were related to increased risk with HRs>1 (Figure 2A). Then, the three filtered genes were further included in the least absolute shrinkage and selection operator (LASSO) to eliminate the overlapping problem (Figures 2B,C). Afterward, these three candidate genes were conducted to build a prognostic signature by performing a multivariate Cox regression analysis, and detailed information is shown in Supplementary Table S2. The risk score = (0.05951 * expression of *LDHD*) + (0.30230 * expression of *LYRM7*) + (−0.04653 * expression of *PNKD*). We calculated the risk score of each sample based on the three genes’ expression level and their coefficient, and the samples were divided into two-risk subgroups based on the median risk score (Figure 2D). The principal component analysis (PCA) proved that samples with two risk scores were divided into two groups (Figure 2E). The risk score distribution and survival status are shown in Figures 2F,G. As the risk score increases, the risk of death increases, and the OS time decreases. Kaplan–Meier survival curves analyses found that the low-risk subgroup had a longer OS than the high-risk group (*p* = 0.0064; Figure 3A). Subsequently, the time-dependent ROC curve analyses were generated to estimate the predictive ability of the prognostic signature (Figure 3C). The AUC of the prognostic signature was 0.692, which manifested that the lactate metabolism-related signature had moderate predictive power. To assess the robustness of the signature, we performed a survival analysis using the validation cohort from the GEO dataset (GSE53031). The patients from the validation cohort were divided into the high-risk and low-risk groups by the median value of risk scores calculated with the same formula from TCGA cohort. Consistent with our previous analysis, patients in the high-risk group had a poorer prognosis than those in the low-risk group (*p* = 0.014; Figure 3B). The AUC of the prognostic signature was 0.731 (Figure 3D).

### Independent prognostic value of the risk model

**FIGURE 4:**
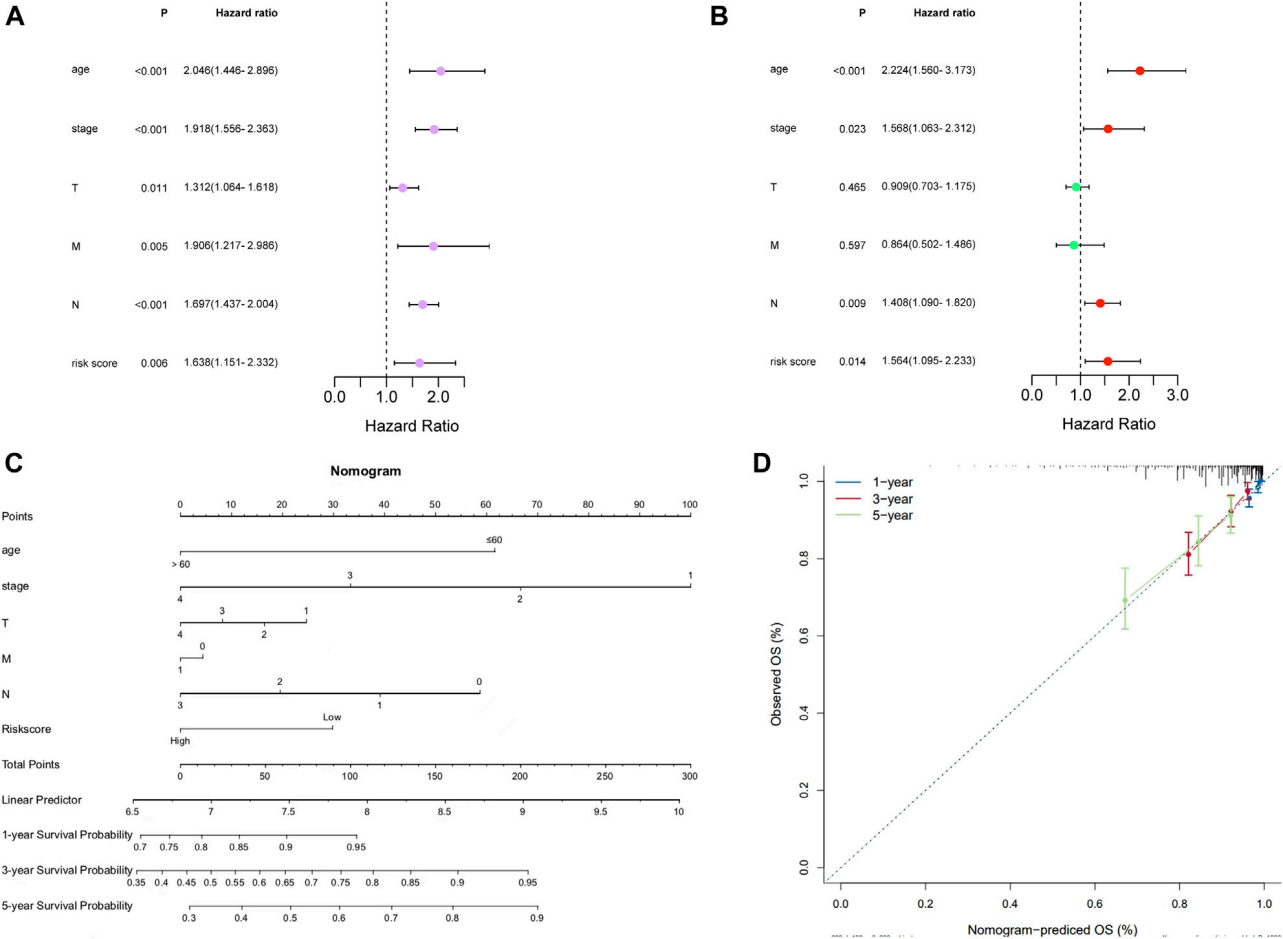
Independent prognostic value of the risk model in TCGA cohort. **(A)** Forest of Univariate analysis. **(B)** Forest of multivariate analysis. **(C)** Nomogram to predict the 1-, 3-, and 5-year overall survival rate of BC patients. **(D)** Calibration curves of the nomogram for 1-, 3-, and 5-year OS prediction.

To further explore whether the prognostic signature can independently predict the survival rate of BC, univariate and multivariate Cox regression analyses were generated. The univariate Cox regression analysis showed that the risk score was an independent factor predicting poor OS in BC (HR = 1.6380, 95% CI: 1.1508–2.3316; Figure 4A). The multivariate analysis obtained the same conclusion, and the risk score was an independent prognostic factor (HR = 1.5637, 95% CI: 1.0952–2.2326; Figure 4B) after adjusting other clinical features, including age, tumor stage, T, N, and M.

### Construction of the nomogram

We constructed a nomogram for the prognosis of BC combined with clinicopathological characteristics such as age, stage, pT, pN, and pM. The prognostic nomogram can predict tumors at 1, 3, and 5 years (Figure 4C), and the calibration curve confirms the accuracy of the nomogram in predicting survival prognosis (Figure 4D).

### Functional enrichment analysis of the signature

**FIGURE 5:**
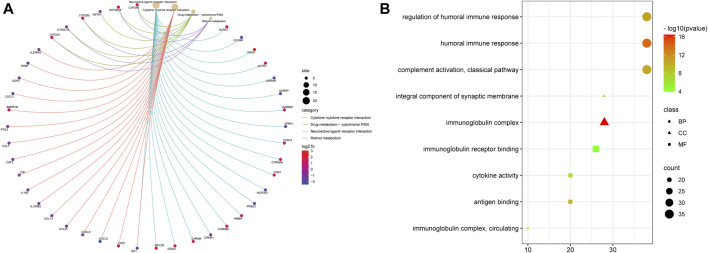
Functional analysis based on the DEGs between the two risk groups. **(A)** Bubble graph for GO enrichment (the bigger bubble means more genes are enriched, and the increasing depth of red means the differences were more obvious. **(B)** Cnetplot for KEGG pathways.

To further study the difference between functions and pathways in the two risk subgroups of this risk model, we extracted 1,260 DEGs from two risk groups by conducting the “limma” R package with specific criteria (|log2FC| ≥ 1 and FDR <0.05). Among them, 381 DEGs were downregulated, and 879 DEGs were upregulated. Then we utilized the obtained DEGs for GO and KEGG analyses. We found that those 1,260 DEGs were mainly correlated with neuroactive ligand–receptor interaction, cytokine–cytokine receptor interaction, and drug metabolism–cytochrome P450 by KEGG analysis (Figure 5A). The GO study showed that those DEGs were mainly related to humoral immune response, regulation of humoral immune response, immunoglobulin complex, an integral component of synaptic membrane, immunoglobulin receptor binding, and antigen-binding.

### Comparison of immune activity between the two subgroups

**FIGURE 6:**
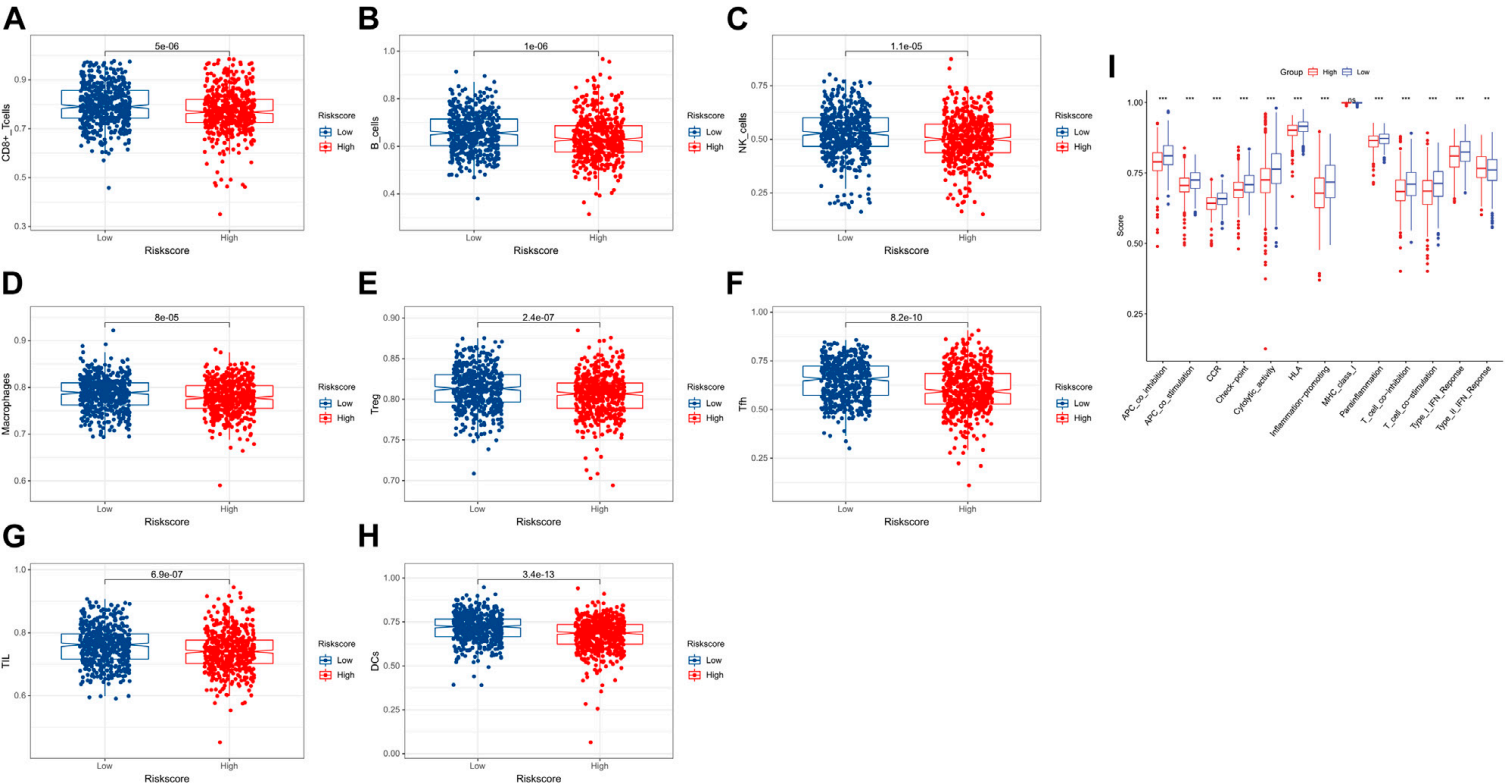
Comparison of the ssGSEA scores for immune cells and immune pathways. **(A–H)** Comparison of the two risk groups for immune cells. **(I)** Comparison of the two risk groups for immune pathways.

It is worth noting that most cell functions are related to immune response. Therefore, immune cells and pathways were further compared between the two risk groups. Based on the function analysis, we compared the enrichment scores of 8 critical immune cells and 13 immune-related pathways between the low- and high-risk subgroups by conducting the single-sample gene set enrichment analysis (ssGSEA). The result showed that the 8 types of immune cells (CD8 cells, B cells, NK cells, macrophages, Tfh, TIL, Treg, and DCs) were significantly correlated with the lactate metabolism-related prognostic signature (*p* < 0.001), and the low-risk group had higher levels of infiltration of immune cells (Figures 6A–H). When comparing the immune-related pathways, similar conclusions were drawn. All tracks except MHC_class_I proved to be statistically significant (*p* < 0.001) and the low-risk group had higher immune-related pathway activity than the high-risk group (Figure 6I).

### Risk signature was associated with immune checkpoints and tumor biomarkers

**FIGURE 7:**
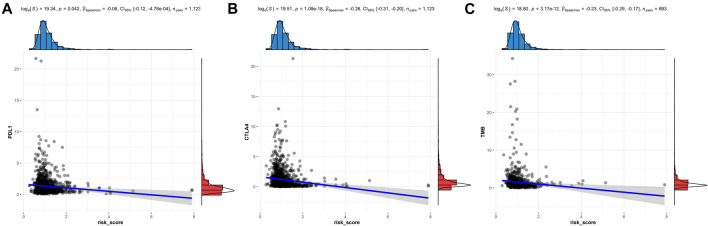
Immune checkpoints and TMB analysis of the LMRG signature. The risk score is related to PD-L1 **(A)**, CTLA4 **(B)**, and TMB **(C)**.

Previous results indicated the relationship between the lactate metabolism-related signature and the immune response. Overexpression of immune checkpoints in tumor cells has been proven to be an immune escape mechanism, and many innovative drugs targeting immune checkpoints have become a new direction for immunotherapy. Therefore, PD-L1 and CTLA-4 as important immune checkpoints, were compared in the risk signature (Figures 7A,B). We found a negative association between the risk score and the expression level of checkpoints (PD-L1 and CTLA4). The TMB, as a tumor biomarker, can be used to predict the efficacy of immunotherapy for BC. So the TMB was compared in the risk signature. The results showed that the risk scores were negatively associated with the expression level of the TMB (Figure 7C).

### Lactate metabolism prognostic genes were associated with tumor immune infiltration in BC

**FIGURE 8:**
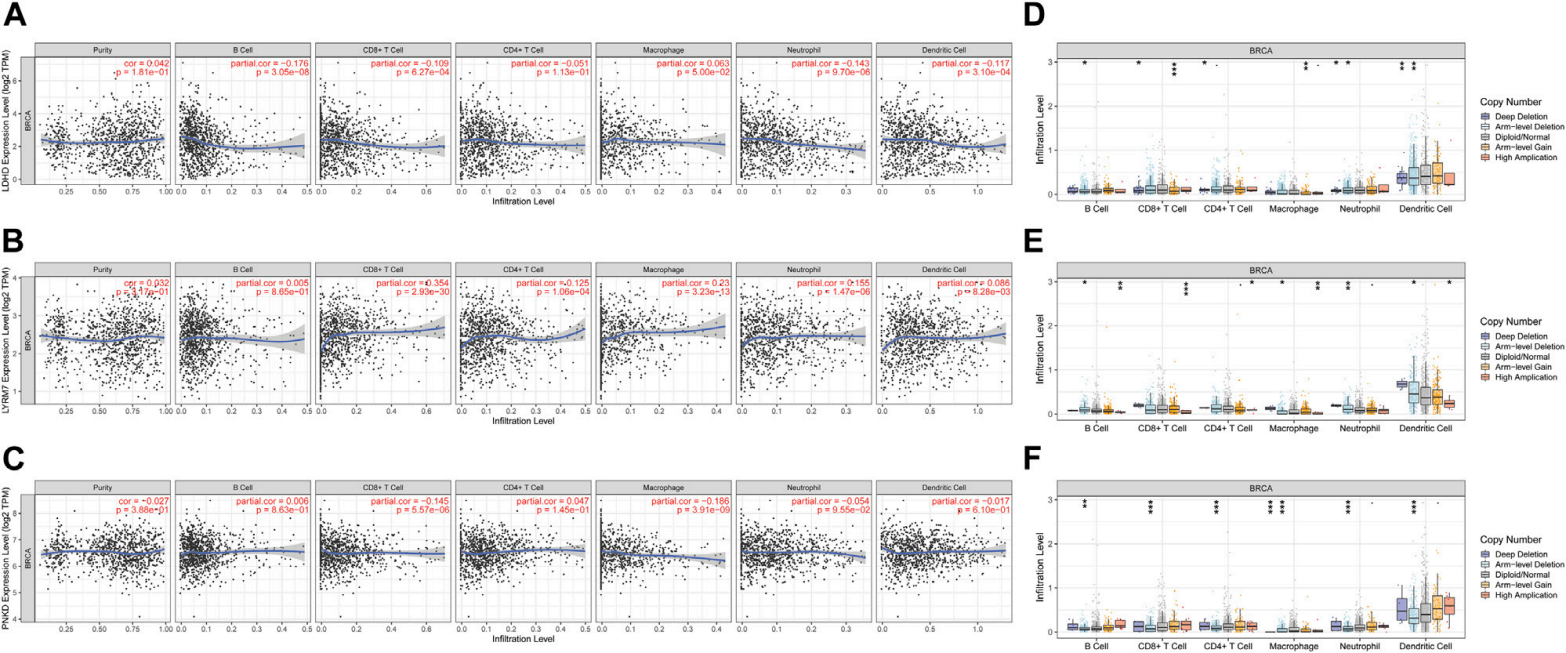
Association between three prognostic genes and immune infiltration in BC. The association between the immune cells and the expression of LDHD **(A)**, LYRM7 **(B)**, and PNKD **(C)**. Relationship between the mutants of three model-related genes and immune cell infiltration LDHD **(D)**, LYRM7 **(E)**, and PNKD **(F)**. *p < 0.05, **p < 0.01, and ***p < 0.001.

Lactate metabolism plays an essential role in the tumor microenvironment. Therefore, we also explored the correlation between the expression of LMRGs (*LDHD, LYRM7,* and *PNKD*) and BC immune infiltration (Figures 8A–C). The results showed that the LMRGs’ expression was correlated with most immune cells (Supplementary Table S3). We also utilized the TIMER database to further explore the relationship between immune cell infiltration and somatic copy number changes (CNAS) of lactate metabolism prognostic genes (Table 1). The results illuminated that arm-level deletion and arm-level gain of *LDHD* were associated with breast cancer immune cell infiltration (Figure 8D). High amplification of *LYRM7* was associated with breast cancer immune cell infiltration (Figure 8E). Arm-level deletion of *PNKD* was associated with breast cancer immune cell infiltration in BC (Figure 8F).

**TABLE 1:** Major mutation types affecting cell infiltration.

Variable	LDHD		LYRM7		PNKD	
	Cna level	*p*	Cna level	*p*	Cna level	*p*
B cell	Arm-level deletion	0.039	High amplication	0.004	Arm-level deletion	0.004
CD8^+^ T cell	Arm-level gain	0.001	High amplication	0.001	Arm-level deletion	<0.001
CD4^+^ T cell	Deep deletion	0.022	High amplication	0.034	Arm-level deletion	<0.001
Macrophage	Arm-level gain	0.009	High amplication	0.007	Deep deletion	<0.001
Neutrophil	Arm-level deletion	0.019	Arm-level deletion	0.014	Arm-level deletion	<0.001
Dendritic cell	Arm-level deletion	0.005	High amplication	0.016	Arm-level deletion	<0.001

Cna, copy number alterations.

### Construction of a network of mRNA–miRNA

**FIGURE 9:**
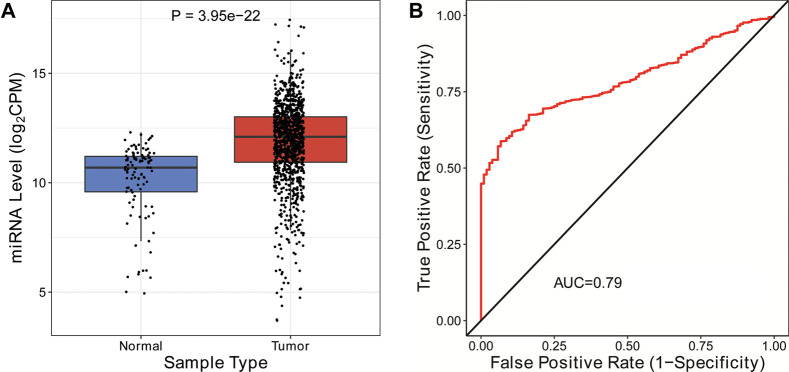
Construction of mRNA-miRNA network. **(A)** Expression of has-mir-203a-3p in BC. **(B)** ROC curves for the OS of the expression of has-mir-203a-3p in BC.

*LDHD* and *LYRM7* are more closely related to prognosis (*p* < 0.05) in the multivariate Cox regression analysis, so we constructed an mRNA–miRNA network to further study the potential molecular mechanism of *LDHD* and *LYRM7* in BC. We found that miR-203a-3p acts as a targeted mRNA to bind *LDHD* and *LYRM7* according to the TargetScan database. Further study showed that miR-203a-3p was upregulated in BC (Figure 9A; *p* = 3.95e-22). Moreover, the ROC indicated that miR-203a-3p could be utilized to distinguish between BC tissue and adjacent breast tissue by data analysis in the CancerMIRNome dataset (http://bioinfo.jialab-ucr.org/CancerMIRNome) (AUC = 0.79; Figure 9B).

## Discussion

BC is the leading cause of female deaths, and there is an urgent need for robust prognostic signatures to be stratified to achieve precise treatment. Increasing evidence shows that lactate metabolism is related to tumor maintenance, progression, and immunosuppression. Excessive lactate can alter the normal function of immune cells, which can encourage cancer cell proliferation (de Bari and Atlante, 2018; Feichtinger and Lang, 2019). Accordingly, we evaluated the prognosis of LMRGs and established a prognostic signature to provide a more accurate treatment strategy for BC.

Our prognostic signature consisted of three LMRGs (*LDHD, LYRM7,* and *PNKD*). They all play various roles in tumor progression. The source of lactate is believed to be via conversion of pyruvate by lactate dehydrogenase (LDH) in the last step of glycolysis (Mack et al., 2017). *LDHD* is a member of the LDH gene family; changes in LDHD gene expression affect the process of lactate metabolism through the glycolytic pathway. One study has confirmed its correlation with kidney cancer prognosis (Wang et al., 2018). Lidia de Bari believed that the expression of *LDHD* is higher in prostate cancer cells than in normal tissues (de Bari et al., 2013). However, its role in breast cancer is unclear due to the lack of data. *LYRM7* encodes the mitochondrial LYR motif-containing protein 7, a part of the mitochondrial respiratory chain complex III (CIII). The defect in CIII can cause a rare mitochondrial disorder, which affects the process of lactate metabolism (Fernández-Vizarra and Zeviani, 2015). *PN*KD is a functional gene known as paroxysmal nonkinesigenic dyskinesia, which is overexpressed in many solid malignancies, such as chronic myelogenous leukemia, breast cancer, and hepatoma. PNKD can lead to tumor cell differentiation, accelerates metastasis, and initiates malignant transformation (Wang et al., 2018).

We further explore the function analysis among the two risk subgroups of the prognostic signature. The results showed that the signature was significantly related to immune response. As we all know, the immune microenvironment plays an essential role in the development and maintenance of cancer (Itoh et al., 2018). Therefore, we study the relationship between the lactate metabolism-related prognostic signature and the immune microenvironment. Different degrees of immune cell infiltration (CD8^+^ T cells, B cells, NK, macrophages, Treg, Tfh, TIL, and DCs) are related to the prognostic signature and LMRGs. CD8^+^ T cells are the most critical tumor killer in the anti-cancer immune response and play a decisive role in anti-cancer immunotherapy (Crispin and Tsokos, 2020). Antigens derived from tumor tissues can activate CD8^+^ T cells to produce anti-cancer responses, improving pore formation in target tumor cell membranes and subsequent target-cell killing by secreting cathepsin C, perforin, and granulysin to fusion target cell membranes. Moreover, CD8^+^ T cells and Treg play a synergistic role in the anti-tumor process (Raskov et al., 2021). The heterogeneity of B cells in tumors makes the impact of these cells on tumors controversial. A retrospective study believed that high expression of B cells and immunoglobulin genes is beneficial to prolonging survival (Olkhanud et al., 2011). However, some studies have linked B cells to the poor prognosis of BC (Tower et al., 2019). NK cells have both adaptive and innate immune properties. NK cells destroy tumors by producing pro-inflammatory cytokines and recruiting other immune cells. They also play a powerful role in cancer immune surveillance. Growing evidence shows that NK cells become dysfunctional during BC (Slattery et al., 2021). Therefore, solving NK cell dysfunction may become new cellular immunotherapy. M1 macrophages can activate the tumor immune response to produce the tumor-killing effect. On the contrary, M2 macrophages will help tumor cells evade immune surveillance, which is not conducive to patient survival (Moradi-Chaleshtori et al., 2021). Tfh promotes tumor immune response by germinal center formation, activating B cells, and antibody class switching (Jogdand et al., 2016). To sum up, immune cells play various roles in the development of tumors. Immune cell infiltration also determines the probability of response to cancer immunotherapies (van der Leun et al., 2020). In our study, immune cell infiltration in the low-risk group is more abundant, which indicates more sensitivity to immunotherapy.

Applying immune checkpoint inhibitors (ICI) to cancer is a milestone on the road to anti-tumor immunotherapy. ICI therapy, whether as a monotherapy or a combination therapy, has become the first-line treatment option for many types of tumors (Zhou et al., 2021). Among them, anti-CTLA-4 and anti-PD-1/PD-L1 have been approved by the European Medicines Agency (EMA) and Food and Drug Administration (FDA), and they have been applied to various malignant tumors. Tumor cells continuously activate the PD-1/PD-L1 signaling pathway by overexpressing PD-L1, triggering different immunosuppressive mechanisms. PD-1/PD-L1 has been considered the target of anti-cancer immunotherapy, and various monoclonal antibody drugs targeting the PD1/PD-L1 signaling pathway have achieved effective outcomes in anti-cancer therapies. The monoclonal antibody mainly interferes with the PD-1/PD-L1 signaling pathway in three aspects: 1) suppressing transcription and translation of PD-1/PD-L1, 2) promoting degradation of the PD-1/PD-L1 protein, and 3) inhibiting the direct combination of PD-1 and PD-L1 (Wu et al., 2021). CTLA-4 is an essential stimulatory receptor that regulates T-cell activation, which can reduce or eliminate immune cell function and encourage tumor cells to escape immune surveillance. Inhibition of CTLA-4 can eliminate immune tolerance and tumor cell proliferation (Kern and Panis, 2021). The TMB represents the number of mutations per megabase (Mut/Mb) in specific cancer. TMB, as the most widely used immunotherapy biomarker to identify populations, predicts better immunotherapy efficacy with higher values (Dong et al., 2021). In our study, the higher values of LMRG risk score are positively related to immune checkpoints and TMB, which shows that it is more sensitive to immunotherapy. Therefore, we can use this prognostic signature to stratify BC patients receiving immunotherapy.

We also developed an mRNA–miRNA network, identifying a miR-203a-3p/LDHD/LYRM7 regulatory axis. Numerous studies have shown that miR-203a-3p plays different roles in various cancers. The higher expression of miR-203a-3p has been detected in colorectal and hepatocellular carcinoma (Huo et al., 2017; Xu et al., 2021). By contrast, the lower expression of miR-203a-3p was detected in esophageal cancer, non-small-cell lung carcinoma, and gastric cancer (Liu et al., 2016; Wang et al., 2018; Liang et al., 2020). Earlier studies have shown that the expression of miR-203a-3p in BC tissues is significantly higher than in normal tissues, which aligns with our research conclusion. In addition, it was detected that upregulated miR-203a-3p was related to age, PR-negative, and ER-negative BC tissue, and it may enhance the oncogenesis and development of BC (Cai et al., 2018). No matter whether in our study or previous research, no prognostic value was observed. But miR-203a-3p could be utilized to distinguish between BC tissue and adjacent breast tissue, which was related to clinical features. Further study should be conducted to verify this result.

Our research draws the following conclusions: 1) we can get the risk score based on the prognostic signature, and then use the nomogram to predict the overall survival rate of patients. 2) The risk score can estimate the patient’s immune status and stratify the patient for immunotherapy. 3) LMRGs are significantly related to the immune response, which provides insights for future research. However, our research also has limitations. First, we were unable to conduct *in vivo* or *in vitro* experiments to verify our research because of the limitation of conditions. Second, the effect of clinical immunotherapy cannot be compared with the model due to data limitations. Third, the area under the AUC curve was not very significant, suggesting that our model had only indicated a nine-gene signature which had a moderate predictive ability. Those challenges will motivate us to continue to explore. Our study also has the advantage that it is the first breast cancer-related model to be constructed using genes related to lactate metabolism, and we investigated in depth the relevance of the model to the immune microenvironment. In addition, our lactate metabolism-related genes are well sourced, mainly from the Molecular Signature Database and published reviews. We believe our study may provide new insight for future anti-cancer therapies.

## Conclusion

In conclusion, a novel lactate metabolism-related prognostic signature was constructed and can be used to predict the prognosis of BC patients. In addition, the signature is closely related to the immune microenvironment, which may provide new insight for future anti-cancer therapies.

## Data Availability

Publicly available datasets were analyzed in this study. These data can be found at: The Cancer Genome Atlas (TCGA) (https://portal.gdc.cancer.gov/); Gene Expression Omnibus (GEO) (https://www.ncbi.nlm.nih.gov/geo/); the Molecular Signature Database v7.4 (MSigDB; https://www.gsea-msigdb.org/gsea/msigdb); and the Tumor Immune Estimation Resource (TIMER; https://cistrome.shinyapps.io/timer/).
