# Supplementary material for: A novel lactate metabolism-related signature predicts prognosis and tumor immune microenvironment of breast cancer
Source: Front Genet. 2022 Sep 7;13:934830. doi: 10.3389/fgene.2022.934830 (PMC9511350; doi:10.3389/fgene.2022.934830)
Supplement: Supplementary file 1 [file Table1.DOCX]

**Supplementary table 1|** Lactate metabolic-related genes of breast cancer.

| Genes | LogFC | P | FDR | Full-names |
| --- | --- | --- | --- | --- |
| ACTN3 | -5.13 | 0.007 | 0.009 | actinin alpha 3 |
| LDHA | 0.55 | < 0.001 | < 0.001 | lactate dehydrogenase A |
| LDHD | -1.48 | < 0.001 | < 0.001 | lactate dehydrogenase D |
| LYRM7 | -0.62 | < 0.001 | < 0.001 | LYR motif containing 7 |
| MIR210 | 1.42 | < 0.001 | < 0.001 | microRNA 210 |
| MYC | -0.99 | < 0.001 | < 0.001 | MYC proto-oncogene |
| PER2 | -1.25 | < 0.001 | < 0.001 | period circadian regulator 2 |
| PFKFB2 | 0.50 | < 0.001 | < 0.001 | 6-phosphofructo-2-kinase/fructose-2,6-biphosphatase 2 |
| PNKD | 1.03 | < 0.001 | < 0.001 | Paroxysmal nonkinesiogenic dyskinesia |
| PTEN | -0.62 | < 0.001 | < 0.001 | phosphatase and tensin homolog |
| SLC16A3 | 2.25 | < 0.001 | < 0.001 | solute carrier family 16 member 3 |
| SLC5A12 | 2.39 | < 0.001 | < 0.001 | solute carrier family 5 member 12 |
