# Supplementary figures and images for: A novel lactate metabolism-related signature predicts prognosis and tumor immune microenvironment of breast cancer
Source: Front Genet. 2022 Sep 7;13:934830. doi: 10.3389/fgene.2022.934830 (PMC9511350; doi:10.3389/fgene.2022.934830)

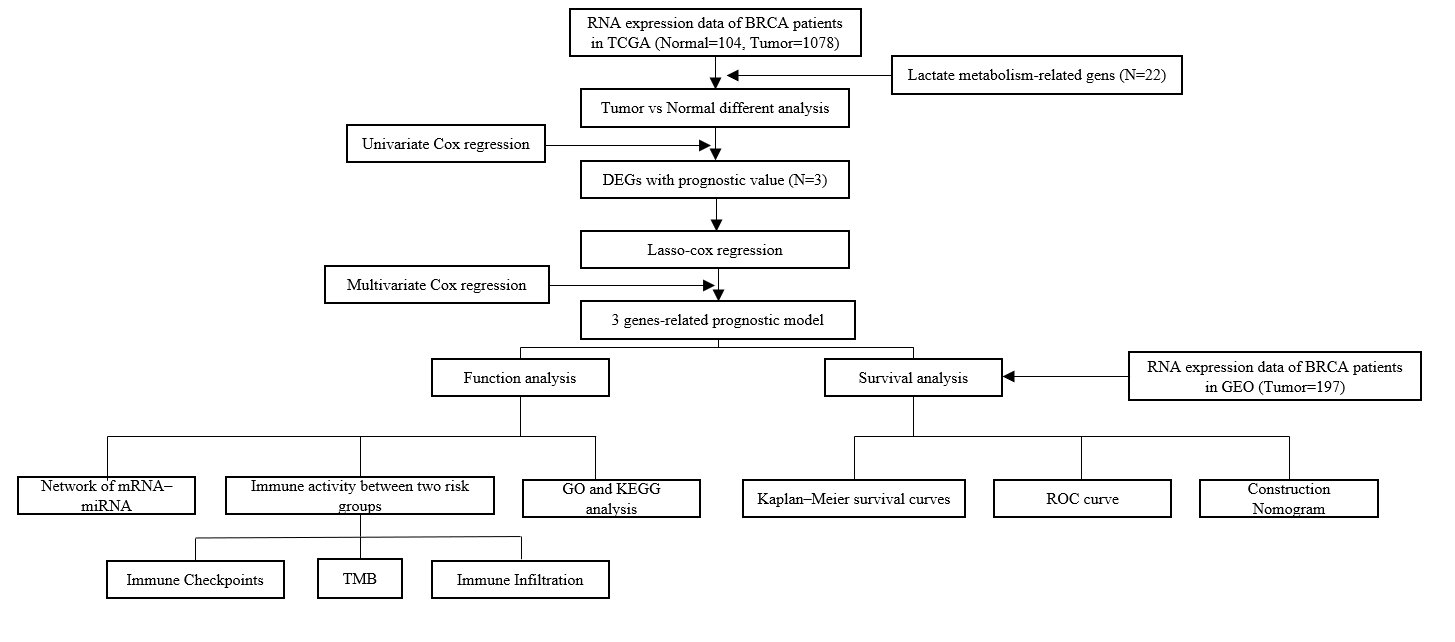

Supplement: Supplementary file 2 [file Image1.JPEG]
