# Supplementary material for: A novel lactate metabolism-related signature predicts prognosis and tumor immune microenvironment of breast cancer
Source: Front Genet. 2022 Sep 7;13:934830. doi: 10.3389/fgene.2022.934830 (PMC9511350; doi:10.3389/fgene.2022.934830)
Supplement: Supplementary file 3 [file Table2.DOCX]

| Gene | Coefficient | *P* vaule |
| --- | --- | --- |
| LDHD | 0.05951 | 0.0463 |
| LYRM7 | 0.30230 | 0.0137 |
| PNKD | -0.04653 | 0.1549 |

**Supplementary table 2|** Three lactate metabolism-related genes of the signature.
