# Supplementary material for: A novel lactate metabolism-related signature predicts prognosis and tumor immune microenvironment of breast cancer
Source: Front Genet. 2022 Sep 7;13:934830. doi: 10.3389/fgene.2022.934830 (PMC9511350; doi:10.3389/fgene.2022.934830)
Supplement: Supplementary file 4 [file Table3.DOCX]

**Supplementary table 3|** Correlation of gene expressions with immune infiltration levels in BC.

| Variable | LDHD | | LYRM7 | | PNKD | |
| --- | --- | --- | --- | --- | --- | --- |
|  | cor | *P* | cor | *P* | cor | *P* |
| B Cell | -0.170 | <0.001 | 0.005 | 0.865 | 0.006 | 0.863 |
| CD8+ T Cell | -0.109 | <0.001 | 0.354 | <0.001 | -0.145 | <0.001 |
| CD4+ T Cell | -0.051 | 0.113 | 0.125 | <0.001 | 0.047 | 0.145 |
| Macrophage | 0.063 | 0.005 | 0.23 | <0.001 | -0.189 | <0.001 |
| Neutrophil | -0.143 | <0.001 | 0.155 | <0.001 | -0.054 | 0.096 |
| Dendritic Cell | -0.117 | <0.001 | 0.086 | <0.001 | -0.017 | 0.610 |

- Cor=correlation
